# Multicenter Randomized Controlled Crossover Trial Comparing Hemodynamic Optimization Against Echocardiographic Optimization of AV and VV Delay of Cardiac Resynchronization Therapy: The BRAVO Trial

**DOI:** 10.1016/j.jcmg.2018.02.014

**Published:** 2019-08

**Authors:** Zachary I. Whinnett, S.M. Afzal Sohaib, Mark Mason, Edward Duncan, Mark Tanner, David Lefroy, Mohamed Al-Obaidi, Sue Ellery, Francisco Leyva-Leon, Tim Betts, Mark Dayer, Paul Foley, Jon Swinburn, Martin Thomas, Raj Khiani, Tom Wong, Zaheer Yousef, Dominic Rogers, Paul R. Kalra, Vignesh Dhileepan, Katherine March, James Howard, Andreas Kyriacou, Jamil Mayet, Prapa Kanagaratnam, Michael Frenneaux, Alun D. Hughes, Darrel P. Francis

**Affiliations:** a Department of Cardiology, International Centre for Circulatory Health, National Heart and Lung Institute, Imperial College London, London, United Kingdom; b Department of Cardiology, Royal Brompton & Harefield NHS Trust, Middlesex, United Kingdom; c Department of Cardiology, Bristol Heart Institute, Bristol, United Kingdom; d Department of Cardiology, Western Sussex Hospitals NHS Foundation Trust, West Sussex, United Kingdom; e Department of Cardiology, Frimley Health, Wexham Park Hospital, Slough, United Kingdom; f Department of Cardiology, Brighton & Sussex University Hospitals NHS Trust, Brighton, United Kingdom; g Department of Cardiology, University Hospitals Birmingham NHS Trust, Birmingham, United Kingdom; h Department of Cardiology, Oxford University Hospitals NHS Trust, Oxford, United Kingdom; i Department of Cardiology, Taunton & Somerset NHS Foundation Trust, United Kingdom; j Department of Cardiology, Great Western Hospitals NHS Foundation Trust, Swindon, United Kingdom; k Department of Cardiology, Royal Berkshire Hospitals NHS Trust, Reading, United Kingdom; l Department of Cardiology, University College London Hospitals NHS Foundation Trust, London, United Kingdom; m Department of Cardiology, Milton Keynes Hospital NHS Trust, Milton Keynes, United Kingdom; n Department of Cardiology, University Hospital of Wales, Cardiff, United Kingdom; o Department of Cardiology, Royal Free Hospital NHS Trust, London, United Kingdom; p Department of Cardiology, Portsmouth Hospitals NHS Trust, Portsmouth, United Kingdom; q Department of Cardiology, Sheffield Teaching Hospitals NHS Foundation Trust, Sheffield, United Kingdom; r Department of Cardiology, Institute of Medical Sciences, University of Aberdeen Foresterhill, Aberdeen, United Kingdom; s Department of Cardiology, Institute of Cardiovascular Sciences, University College London, London, United Kingdom

**Keywords:** biventricular pacing, cardiac resynchronization therapy, echocardiographic optimization, heart failure, hemodynamic optimization, optimization, AF, atrial fibrillation, AV, atrioventricular, CI, confidence interval, CRT, cardiac resynchronization therapy, LV, left ventricular, NT-proBNP, N-terminal pro–B-type natriuretic peptide, VV, ventriculoventricular

## Abstract

**Objectives:**

BRAVO (British Randomized Controlled Trial of AV and VV Optimization) is a multicenter, randomized, crossover, noninferiority trial comparing echocardiographic optimization of atrioventricular (AV) and interventricular delay with a noninvasive blood pressure method.

**Background:**

Cardiac resynchronization therapy including AV delay optimization confers clinical benefit, but the optimization requires time and expertise to perform.

**Methods:**

This study randomized patients to echocardiographic optimization or hemodynamic optimization using multiple-replicate beat-by-beat noninvasive blood pressure at baseline; after 6 months, participants were crossed over to the other optimization arm of the trial. The primary outcome was exercise capacity, quantified as peak exercise oxygen uptake. Secondary outcome measures were echocardiographic left ventricular (LV) remodeling, quality-of-life scores, and N-terminal pro–B-type natriuretic peptide.

**Results:**

A total of 401 patients were enrolled, the median age was 69 years, 78% of patients were men, and the New York Heart Association functional class was II in 84% and III in 16%. The primary endpoint, peak oxygen uptake, met the criterion for noninferiority (p_noninferiority_ = 0.0001), with no significant difference between the hemodynamically optimized arm and echocardiographically optimized arm of the trial (mean difference 0.1 ml/kg/min). Secondary endpoints for noninferiority were also met for symptoms (mean difference in Minnesota score 1; p_noninferiority_ = 0.002) and hormonal changes (mean change in N-terminal pro–B-type natriuretic peptide -10 pg/ml; p_noninferiority_ = 0.002). There was no significant difference in LV size (mean change in LV systolic dimension 1 mm; p_noninferiority_ < 0.001; LV diastolic dimension 0 mm; p_noninferiority_ <0.001). In 30% of patients the AV delay identified as optimal was more than 20 ms from the nominal setting of 120 ms.

**Conclusions:**

Optimization of cardiac resynchronization therapy devices by using noninvasive blood pressure is noninferior to echocardiographic optimization. Therefore, noninvasive hemodynamic optimization is an acceptable alternative that has the potential to be automated and thus more easily implemented. (British Randomized Controlled Trial of AV and VV Optimization [BRAVO]; NCT01258829)

## Graphical abstract

**Figure:**
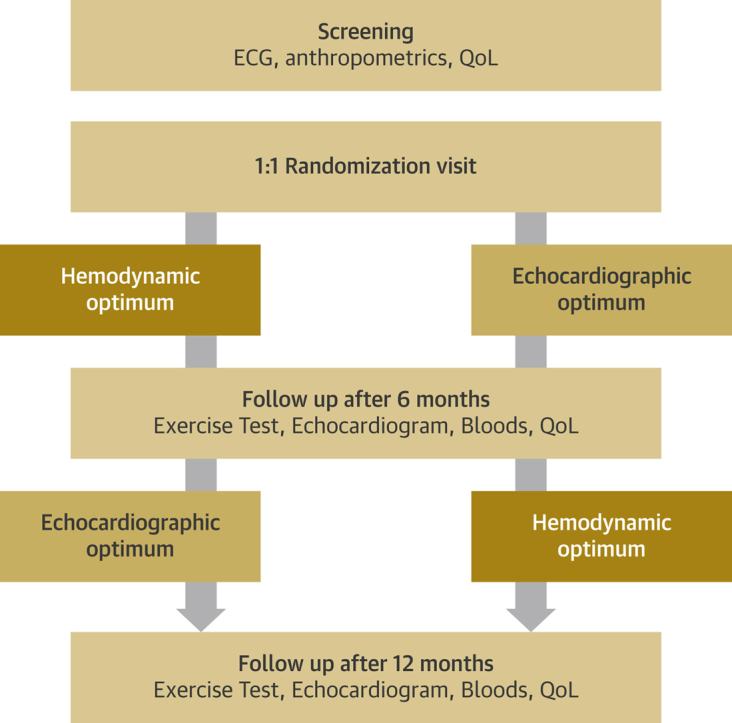

Delivering cardiac resynchronization therapy (CRT) to appropriately selected patients causes immediate improvements in cardiac function. The early studies observed immediate improvements in hemodynamic parameters 1, 2, 3, including peak rates of rise of intraventricular pressure (4), stroke volume (5), and blood pressure 4, 6. Subsequent studies revealed improvements in exercise capacity, left ventricular (LV) volumes, quality of life 7, 8, 9, and N-terminal pro–B-type natriuretic peptide (NT-proBNP) values (10). Finally, large, randomized, long-term studies demonstrated reductions in hospitalizations and mortality 11, 12.

The beneficial effects of CRT stem ultimately from the changes in timing of cardiac activation. The landmark CARE-HF (CArdiac REsynchronisation in Heart Failure) trial performed atrioventricular (AV) delay optimization after device implantation by using echocardiography. Echocardiography remains the most commonly recommended method for optimization 13, 14. In this process the AV delay is set to maximize separation of the E and A waves on transmitral Doppler imaging. The precise AV and ventriculoventricular (VV) delays that maximize hemodynamic measurements vary among patients, perhaps because of the complexity of the disease and anatomic variations in lead position 15, 16.

In current clinical practice, however, many patients do not undergo an echocardiographic optimization process, partly because of shortage of skilled staff time. Only 40% of physicians perform any form of optimization (13). There was initially doubt over the benefit of optimizing CRT (17), but there have been recent encouraging results on clinical outcomes using the AdaptivCRT algorithm (Medtronic, Minneapolis, Minnesota) (18) and methods dependent on implanted hemodynamic sensors (SonR, LivaNova, London, United Kingdom) (19). However, each of these algorithms is limited to a single manufacturer. The ideal alternative to the time- and labor-intensive echocardiographic method, with reproducibility that can be challenging (20), would be a manufacturer-independent and fully automatable method.

Instead of Doppler findings, an alternative target for optimization is blood pressure, which does not require expert judgment and therefore can be automated to accelerate analysis and save resources. It is important to take steps to minimize noise, and we have therefore developed an acquisition protocol that involves taking multiple repeated measurements between the tested setting and a reference setting (16). We have previously shown that when systolic blood pressure is used, it is most efficient to sample immediately after the change in pacemaker setting. This is because even if no changes are made, the pressure tends to change away from the starting value with the passage of time in response to spontaneous physiological processes. In addition, when settings are changed to improve cardiac output there is a resulting reflex fall in peripheral resistance that returns the blood pressure toward the mean, even though improvements in cardiac output remain (15). It is therefore better to sample blood pressure before this occurs.

The BRAVO (British Randomized Controlled Trial of AV and VV Optimization) trial tested the hypothesis that physiological optimization of AV and VV delay would be noninferior to echocardiographic optimization.

## Methods

### Study design

The design (21) was a crossover trial that was prospective, randomized, and open with blinded evaluation of endpoints. Analysis of cardiopulmonary exercise test data, echocardiography, and blood results was performed by investigators blinded to the study arm.

**Figure 1:**
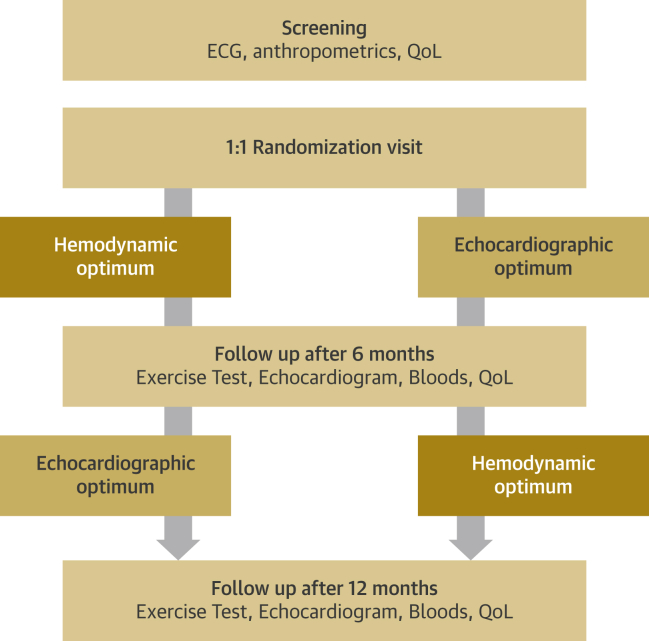
Flowchart of Study Design Patients visited 4 times and underwent 2 atrioventricular and interventricular delay optimizations according to echocardiographic and hemodynamic protocols. ECG = electrocardiography; QoL = quality of life.

Patients were recruited from 19 centers in the United Kingdom. Patients were randomly allocated to an optimization method using an online system. They were followed up for 6 months and then crossed over to the other optimization method for a further 6 months of follow-up (Figure 1).

### Inclusion and exclusion criteria

Study inclusion and exclusion criteria are shown in Table 1.

**Table 1:** Inclusion and Exclusion Criteria

Inclusion Criteria	Exclusion Criteria
Previous diagnosis of chronic heart failure	Major cardiovascular event within 6 weeks before enrollment
Cardiac resynchronization therapy device implanted at least 6 months before enrollment	Uncontrolled hypertension
History of symptomatic congestive heart failure (NYHA functional class II–IV)	Inability to walk on treadmill
Prior ejection fraction <40% or documented moderate to severely impaired systolic dysfunction	
Stable medical therapy for heart failure	
>90% biventricular pacing	

NYHA = New York Heart Association.

### Optimization of AV and VV delay

We performed echocardiographic optimization of the AV delay using Doppler echocardiography of transmitral flow by using the iterative method as used in the CARE-HF trial (22). VV delay optimization was performed by maximizing aortic outflow tract aortic Doppler measurements.

**Figure 2:**
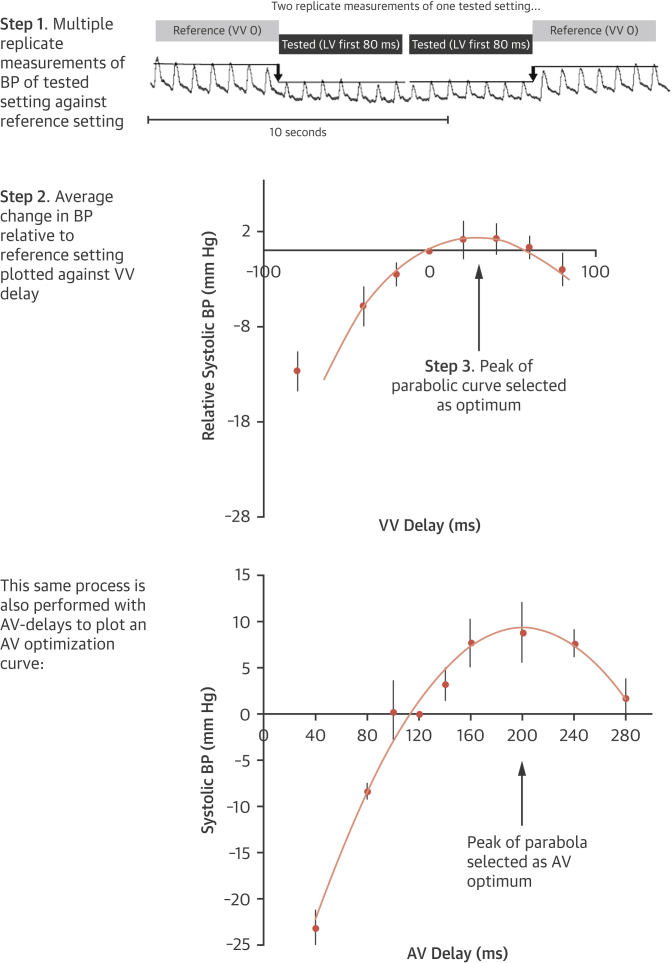
Simplified Schematic of Hemodynamic Optimization Method Continuous noninvasive beat-to-beat measurements are made through the Finometer (Finapres Medical Systems, Amsterdam, the Netherlands). Multiple alternations are carried out between a tested atrioventricular (AV) or ventriculoventricular (VV) delay and reference AV or VV delay. Blood pressures (BPs) before and after a transition in pacing state are measured as an average of 8 to 10 beats, as previously described (16). The average change in BP is plotted against AV or VV delay to fit a curve. The peak of the curve is used to select the optimum. LV = left ventricular.

Hemodynamic optimization of AV and VV delay was performed using multibeat averages acquired through noninvasive blood pressure measured using the Finometer device (Finapres Medical Systems, Amsterdam, the Netherlands). To obtain a narrow confidence interval (CI) we used a specific algorithm (23). This performs multiple alternations between a tested and reference AV delay and calculates the mean relative change in systolic blood pressure. It closely mirrors invasive optimization (24). We first calculated the AV optimum, and then we determined the VV optimum at that AV delay. Some previous studies have used LV dP/dt max as a target for maximization. The BRAVO trial used systolic blood pressure because this can be acquired noninvasively or invasively with equal precision, and it reflects the external consequences of cardiac function. We have previously shown that this method is highly reproducible (25). Its noninvasive nature permits large numbers of replicates, which narrow the CI of the estimated optimum (26). Each hemodynamic optimization (Figure 2) took approximately 20 min, with a further 10 min for off-line analysis. This time could be shortened in the future if the process were automated. When patients were in atrial fibrillation (AF), only VV optimization was performed. Patients with AF were included because if cardiac resynchronization indeed works by resynchronizing the ventricles, as is generally supposed, then the interventricular timing should continue to be as important in AF as it is in sinus rhythm.

### Endpoints

The primary endpoint was objective exercise capacity defined as peak oxygen uptake on cardiopulmonary exercise testing (27). To ensure standardization, all exercise tests were carried out at 1 of 2 sites that are experienced in performing this testing. Secondary outcome measures were LV reverse remodeling, as assessed by echocardiographic LV dimensions (LV end-diastolic dimension and LV end-systolic dimension), NT-proBNP, and quality of life, assessed using standardized scores for heart failure: the 36-Item Short Form Health Survey version 2 (28) and the Minnesota Living with Heart Failure questionnaire score (29).

### Study conduct and regulatory issues

The study was compliant with good clinical practice guidelines and with the most recent version of the Declaration of Helsinki. The study was approved by the South West London Research Ethics Committee (3), and site-specific assessments were performed for each participating hospital. All patients gave prior written informed consent. The trial was registered with ClinicalTrials.gov (NCT01258829).

### Statistics

Distributions are described by their mean ± SD. NT-proBNP is expressed as log_10_ NT-proBNP because it has a positive skew. Comparison between arms of the trial was performed by paired Student’s *t-*test. Analysis was restricted to patients with before and after data for that variable. Differences between arms of the study are expressed as mean and 95% CI. The noninferiority margin for peak oxygen uptake (primary endpoint) was 0.75 ml/kg/min, for the Minnesota Living with Heart Failure score it was 4 points, for the 36-Item Short Form Health Survey version 2 physical component score it was 8.5, for NT-proBNP it was a fall of 0.062 log units (i.e., approximately a 13% decrease), for LV end-diastolic dimension it was 2 mm, and for LV end-systolic volume it was 2 mm. p_noninferiority_ was calculated for these variables against their respective noninferiority margins.

The study sample size was chosen to have 90% power to detect a margin of equivalence of 0.75 ml/kg/min at the 5% significance level, on the basis of a published reproducibility of 2.4 ml/kg/min (30). On this basis, 177 participants per arm of the trial were required.

## Results

**Figure 3:**
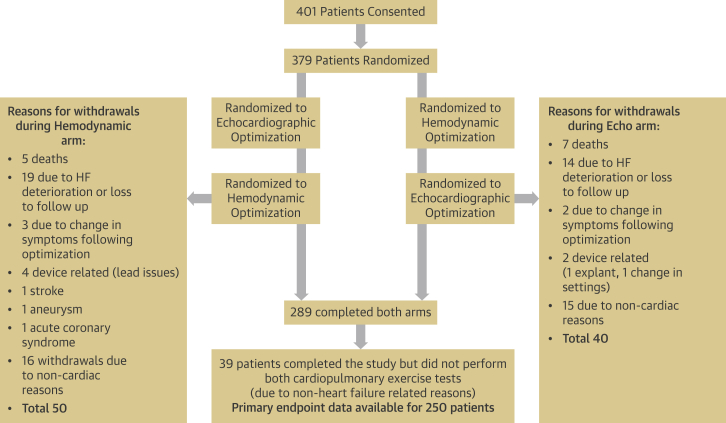
Patient Flow Patients were randomized to either optimization method for 6 months before crossing over to the other arm of the trial for a further 6 months. Investigations performed at each stage are listed. HF = heart failure.

A total of 401 patients met the enrollment criteria and gave informed consent to participate in the BRAVO trial. Baseline characteristics are displayed in Table 2. Patients’ flow and study withdrawals are illustrated in Figure 3. A total of 22 patients did not undergo randomization; 379 patients were randomized, and 48 had AF.

**Table 2:** Baseline Characteristics (n = 401)

Age, yrs	67 ± 12.7
Median	69
Male	78
NHYA functional class	
I	0.3
II	84
III	16
IV	0
CRT-P	36
CRT-D	64
Systolic blood pressure, mm Hg	121 ± 21
Diastolic blood pressure, mm Hg	69 ± 11
Atrial fibrillation	12
Creatinine, μmol/l	112 ± 40
Pharmacotherapy	
ACE inhibitor or angiotensin-receptor blocker	49
Beta-blocker	69
Diuretic agent	56
Mineralocorticoid receptor antagonist	43
Digoxin	19

Values are mean ± SD or %.

ACE = angiotensin-converting enzyme; CRT-D = cardiac resynchronization therapy defibrillator; CRT-P = cardiac resynchronization therapy pacemaker.

There were 12 deaths, 7 during the echocardiographic arm and 5 during the hemodynamic arm. Another 33 patients withdrew from the study because of deterioration in heart failure symptoms or were lost to follow-up, 14 during the echocardiographic arm and 19 during the hemodynamic arm.

Nine patients experienced adverse events unrelated to heart failure that led to withdrawal from the study. Six of these patients had device-related problems (device erosion requiring extraction or loss of LV lead capture, 1 where the settings were changed at another hospital), and 3 patients had other adverse events (1 stroke, 1 myocardial infarction, 1 aneurysm).

A further 31 patients withdrew for specific reasons that were not a deterioration in heart failure. These conditions included musculoskeletal deterioration, peripheral neuropathy, deterioration in balance, torn knee ligaments, depression, terminal cancer, leg ulcers, and stroke. A total of 39 patients completed all the study visits but were unable to complete both exercise tests. See Supplemental Table 1 for baseline characteristics of the participants who did not compete both arms of the trial.

### Comparison of hemodynamic optimization with echocardiographic optimization

#### Cardiopulmonary exercise testing

A total of 250 patients completed the entire 12-month study protocol and performed cardiopulmonary exercise testing after both echocardiographic and hemodynamic optimization.

**Figure 4:**
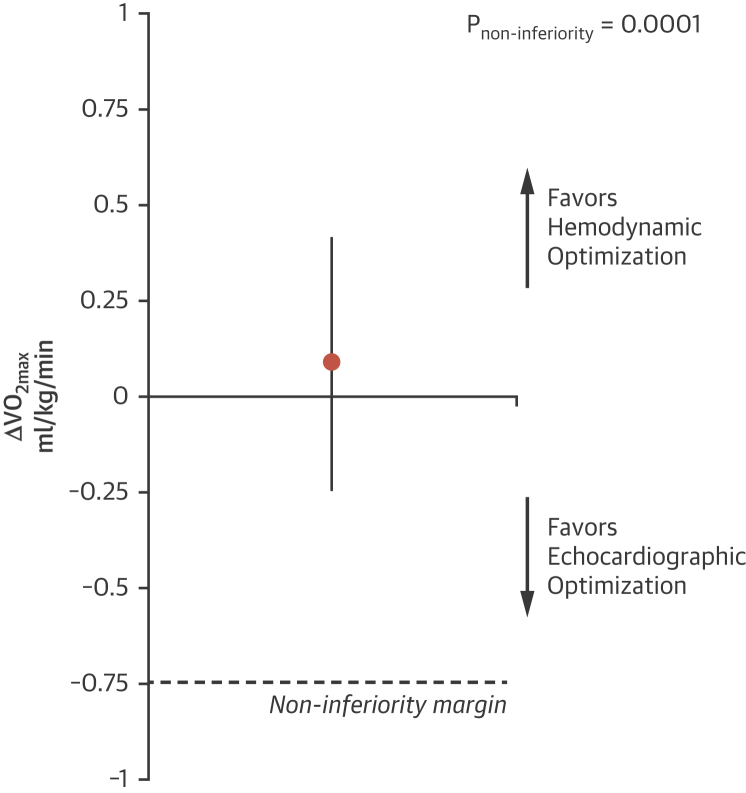
Primary Outcome: Change in ΔVo_2max,_ Shown as Mean With 95% Confidence Interval Hemodynamic optimization using beat-to-beat noninvasive blood pressure was noninferior to the conventional established method of echocardiographic optimization. **Δ**Vo_2max_ = change in peak oxygen uptake.

The results met the primary pre-specified noninferiority criteria (p_noninferiority_ = 0.0001). There was no significant difference in peak oxygen uptake with noninvasive hemodynamic optimization compared with echocardiographic optimization, with a mean difference of 0.1 ml/kg/min (95% CI: −0.25 to +0.41 ml/kg/min) (Figure 4, Table 3).

**Table 3:** Outcome Markers in the 2 Arms of the Study∗

	Echocardiographic Optimization	SD	Hemodynamic Optimization	SD	Difference	n
MLWHFQ score	36	24	37	24	1.0	268
SF36v2 physical component score	38	10	39	10	1.0	269
LVEDD, mm	58	11	58	9	0	284
LVESD, mm	49	12	48	11	1.0	284
Peak VO_2_, ml/kg/min	14.5	4.5	14.6	4.7	0.1	250
NT-proBNP, pg/ml	1,338	2,585	1,348	2,723	10.0	262
Log_10_ NT-proBNP, log_10_ pg/ml	2.8	0.6	2.8	0.6	0	262

LVEDD = left ventricular end-diastolic dimension; LVESD = left ventricular end-systolic dimension; MLWHFQ = Minnesota Living With Heart Failure questionnaire; NT-proBNP = N-terminal pro–B-type natriuretic peptide; SD = standard deviation; SF36v2 = 36-Item Short Form Health Survey version 2; VO_2_ = oxygen uptake.

∗ Scores (with SD) are listed for all the primary and secondary outcomes measures for the study following 6 months of randomization in each arm.

No significant difference was observed in the minute ventilation/carbon dioxide production slope, with a mean difference of 0.3 (95% CI: −0.6 to +1.2; p = 0.20). There was also no difference in exercise duration, with a mean difference of −0.02 min (95% CI: −0.26 to +0.21 min; p = 0.76). See also Supplemental Table 2 for the statistics for order effects.

#### Left ventricular dimension

Differences in end-systolic and end-diastolic dimensions between the 2 arms (n = 284) met criteria for noninferiority, with a mean difference in LV systolic dimension of 1 mm (95% CI: −1.5 to 0.4 mm; p_noninferiority_ <0.0001) and a mean difference in LV diastolic dimension of 0 mm (95% CI: −0.8 to 0.9 mm; p_noninferiority_ <0.0001).

#### Quality of life

##### 36-Item short form health survey version 2

Differences in the symptoms measured by the physical component score of the 36-Item Short Form Health Survey version 2 after 6 months of treatment with hemodynamic optimization compared with 6 months of treatment with echocardiographic optimization met the criteria for noninferiority, with a mean difference of −0.30 (95% CI: −0.4 to 1.0; p_noninferiority_ <0.001; n = 269).

##### Minnesota questionnaire

Differences in the symptoms measured by Minnesota Living With Heart Failure questionnaire score after after 6 months of treatment with hemodynamic optimization compared with 6 months of treatment with echocardiographic optimization met the criteria for noninferiority, with a mean difference of 1.05 (95% CI: −0.72 to 2.90; p_noninferiority_ = 0.002; n = 268).

#### N-terminal pro–B-type natriuretic peptide

Differences in the NT-proBNP after 6 months of treatment with hemodynamic optimization compared with 6 months treatment with echocardiographic optimization met the criteria for noninferiority, with a mean difference of 10 pg/ml (95% CI: **−**180 to 200 pg/ml; p_noninferiority_ <0.002; n = 262).

### Comparison of the AV delay determined as optimal

For the atrial-sensed mode, the AV optimum defined by echocardiography averaged 122 ± 32 ms, and by hemodynamic optimization it was 133 ± 29 ms (difference of 11 ms; p < 0.0001).

### Comparison of VV delay determined as optimal

The VV delay identified as optimal using the hemodynamic method averaged 0 ± 22 ms, and by echocardiography it was LV first 2 ± 34 ms (difference of 2 ms; p = 0.40) (Supplemental Figure 1).

## Discussion

The primary endpoint of exercise capacity was noninferior to hemodynamic optimization compared with echocardiographic optimization. The secondary endpoints of LV dimensions, quality of life, and NT-proBNP were also not significantly different between the 2 methods. These findings suggest that hemodynamic optimization is an acceptable alternative to the established echocardiographic method.

### Hemodynamic optimization

In the trials that showed a survival benefit from biventricular pacing, COMPANION (Comparison of Medical Therapy, Pacing, and Defibrillation in Heart Failure) (12) and CARE-HF (22), AV delay optimization was part of the protocol. In clinical practice, however, echocardiographic optimization has been difficult to incorporate routinely because it requires time and skills in both pacemaker programming and echocardiography, which sometimes require 2 staff members.

Optimizing with blood pressure (hemodynamics) has the advantage of not requiring an expert to keep an echocardiography probe correctly aligned. More importantly, because pressure signals can be quantified automatically, they do not require human expertise to judge echocardiographic Doppler waveforms. In fact, there is no technical barrier to complete automation of the process of hemodynamic optimization. With an appropriately implanted hemodynamic sensor, the process can be carried out automatically and repeatedly, at home.

### AV and VV optimization

This study was a noninferiority study comparing echocardiographic optimization of AV and VV delay with hemodynamic optimization. Some studies have reported that echocardiographic optimization improves outcomes compared with nominal settings 31, 32. However, the SMART-AV (SmartDelay Determined AV optimization: A Comparison to Other AV Delay Methods Used in Cardiac Resynchronization Therapy) trial found no significant effect of optimization of AV delay over nominal settings, after testing both an echocardiographic method and an electrical approach (33).

**Figure 5:**
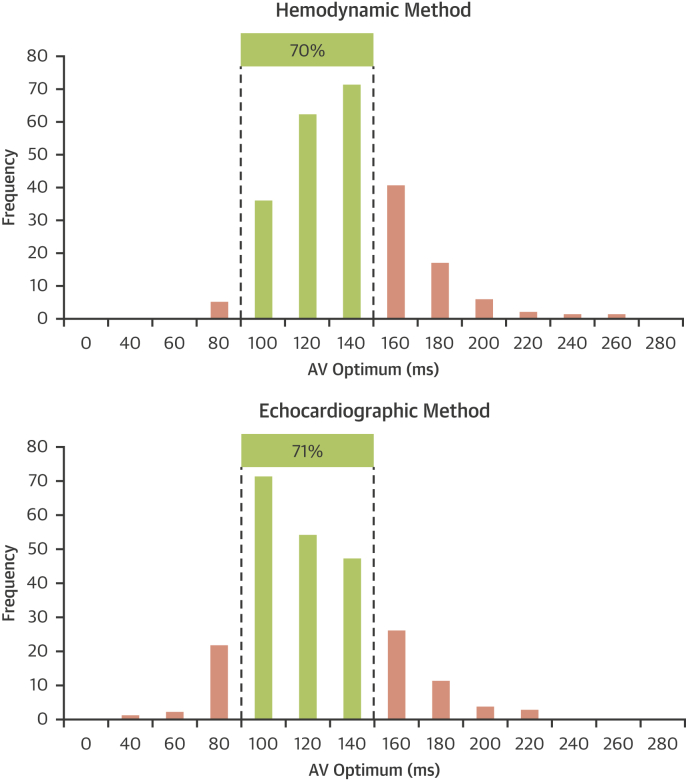
Distribution of AV Delay Identified as Optimal Using the 2 Optimization Methods In approximately one-third of patients, the optimal atrioventricular (AV) delay was found to be more than 40 ms longer or shorter than the commonly used nominal setting of 120 ms. These patients are likely to have the most to gain from AV delay optimization.

A potential explanation for the neutrality of the SMART-AV trial is that in many patients the optimal AV delay is close to 120 ms, a common default value set by manufacturers. In the BRAVO trial, the hemodynamic optimization yielded a sensed AV delay optimum that was within 20 ms of 120 ms in 70% of patients. For echocardiographic optimization, the figure was similar at 71% (Figure 5). Because physiological responses are approximately parabolic near the optimum (34), the curve slopes are most shallow there, so small differences in AV delay near the optimum have only a relatively small effect on physiology (23). This is reflected in the BRAVO trial. Figure 5 shows that the distribution in AV optima between the 2 methods is different, but in both cases ∼70% of the optima are within ±20 ms of 120 ms. It is the ∼30% of patients whose optimal AV delay is more than 20 ms from optimum who are likely to have the most to gain from patient individualized optimization.

The modal AV optima are slightly shorter with echocardiographic than with hemodynamic optimization. However, the difference is small, at 40 ms. Because this occurs at the shallow portion of the AV delay optimization curve, this difference will produce only relatively small changes in cardiac output. These small differences in cardiac output are unlikely to be detected with the clinical outcome measures we used in this study.

The BRAVO trial was not designed to determine whether hemodynamic optimization is superior to nominal settings. To address this question, an adequately powered study would need to be very large because in 70% of patients the nominal AV delay is within 20 ms of optimal. The parabolic nature of the hemodynamic response to adjusting AV delay means that small changes around the optimum have a relatively small effect. Larger differences result in more significant changes in cardiac output because they involve the steeper portion of the AV delay optimization curve.

Hemodynamic optimizations were performed at a higher heart rate using atrial pacing rather than using atrial sensing, to improve signal-to-noise ratio. Rather than performing an optimization during atrial sensing, we programmed the sensed AV delay 60 ms shorter than the AV delay identified as optimal during atrial pacing. It is possible that we would have identified a different optimal-sensed AV delay had we performed optimization during atrial sensing. However, using this protocol we found hemodynamic optimization to be noninferior to echocardiographic optimization.

### Potential practical advantages of hemodynamic optimization

Repeated optimization carried out automatically by the pacemaker was found in the CLEAR (Clinical Evaluation on Advanced Resynchronization) (35) and RESPOND (Clinical Trial of the SonRtip Lead and Automatic AV-VV Optimization Algorithm in the PARADYM RF SonR CRT-D) (19) studies to reduce heart failure hospitalizations in comparison with 1-off optimization. In the CLEAR and RESPOND studies a hemodynamic sensor made the repeated autonomous optimizations possible. In the BRAVO trial only a single optimization was carried out in each arm of the trial. The hemodynamic optimization method, however, does have the potential for automatous repeat optimizations by the device itself if it is combined with an appropriate sensor.

Our previous work has illustrated that the exact choice of sensor is not as important as ensuring that the protocol includes enough repetitions to deliver a reproducible result. The BRAVO trial used a specialized beat-by-beat noninvasive blood pressure monitor, but the same optimum is obtained if a simple pulse oximeter is used (with extra repetitions to combat the extra noise) or if complex invasive arterial monitoring is performed 36, 37

### Study limitations

The BRAVO trial enrolled patients who had undergone CRT implantation procedures at least 6 months prior. The rationale was so the relatively large therapeutic benefit seen in the early phase after CRT implantation would not obscure any differences between the trial arms. Whether the results would have been different if randomization had occurred shortly after device implantation is not known.

In this study, there was a relatively high dropout rate (24% of those randomized); 250 patients completed both exercise tests. Of those participants completing the study, 13% failed to have both exercise tests performed. The numbers of dropouts do, however, appear to be balanced between the groups. The relatively long duration of participation in the study, compared with other CRT studies, may explain the high frequency of dropout. The most common reason for dropout was an inability to carry out exercise testing for noncardiac reasons. Despite the high dropout rate, the study still met the predefined noninferiority threshold. This was because the midpoint value of the result was so close to neutral that the outer limit of the CI was well away from the noninferiority threshold.

This was a heterogeneous, “real-world” group of patients with heart failure who came from a spectrum of centers across the United Kingdom. This study did not have a “run-in period” for optimization of medical therapy because we wanted to test this optimization protocol in real time. Given that this was a crossover study, this was equally applicable to both arms and would not have a net effect on the question under investigation. The real-world nature of this group is also reflected in a perceived discrepancy in symptoms versus physiological markers of heart failure severity. Most patients included were in New York Heart Association functional class II yet had a peak oxygen uptake in the region of 14.5 ml/kg/min, which is more characteristic of patients in New York Heart Association functional class III.

The combined effect of echocardiographic AV and VV delay optimization, compared with nominal settings, has not been assessed in a randomized controlled clinical trial. Our study cannot answer this question because both arms of the trial had optimization performed: it shows that the hemodynamic and echocardiographic approaches are equivalent on the endpoints studied.

Our echocardiographic measurements were taken with the participants in the lateral decubitus position, and the hemodynamic measurements were made with participants semirecumbent. Both measurements were performed with the participants at rest. Ideally, for a direct comparison both should be performed in the same position, but these conventional positions were used for comfort and optimal data acquisition. Echocardiographic measurements were performed only at a resting heart rate because this is the conventional method described for standard echocardiographic optimization.

## Conclusions

The BRAVO trial demonstrates that the physiological effectiveness of hemodynamically selected AV and VV delay is not inferior to that of echocardiographically selected AV and VV delay. Noninvasive hemodynamic optimization is therefore an acceptable alternative to echocardiographic optimization, and it has the potential to be automated and consequently more easily implemented.

Perspectives **COMPETENCY IN MEDICAL KNOWLEDGE:** Noninvasive hemodynamic optimization using beat-to-beat blood pressure measurements is noninferior to widely used echocardiographic methods used for optimization of biventricular pacemaker optimization. This method is much less time consuming and less labor intensive and can provide a much-improved experience for patients.

**TRANSLATIONAL OUTLOOK:** This study did not set out to determine whether hemodynamic optimization confers an additional prognostic benefit. A much larger study would be required to determine this, and it is an area for further study.
