# Supplementary material for: Multicenter Randomized Controlled Crossover Trial Comparing Hemodynamic Optimization Against Echocardiographic Optimization of AV and VV Delay of Cardiac Resynchronization Therapy: The BRAVO Trial
Source: JACC Cardiovasc Imaging. 2019 Aug;12(8):1407–16. doi: 10.1016/j.jcmg.2018.02.014 (PMC6682561; doi:10.1016/j.jcmg.2018.02.014)
Supplement: Supplemental Figure 1 and Supplemental Tables 1 and 2 [file mmc1.docx]

**Supplemental Figure** **1.** Distribution of VV optima using the two different optimization schemes.

0

20

40

60

80

100

120

140

160

180

-80

-60

-40

-20

0

20

40

60

80

Frequency

VV Delay (ms)

Hemodynamic Method

**85%**

0

20

40

60

80

100

120

-80

-60

-40

-20

0

20

40

60

80

Frequency

VV Delay (ms)

Echocardiographic Method

**62**

**%**

**Supplemental Table 1.** Baseline characteristics of the Patients who did not complete both arms

Age (years, mean ± SD)

69 ± 12

median

71

Gender

Male sex (%)

77

NHYA class I (%)

0.0

NHYA class II (%)

80

NHYA class III (%)

20

NHYA class IV (%)

0

CRT-P (%)

43

CRT-D (%)

57

Systolic blood pressure (mmHg, mean ± SD)

116 ± 31

Diastolic blood pressure (mmHg, mean ± SD)

66 ± 15

Creatinine (µmol/L, mean ± SD)

74 ± 64

Use of an ACE inhibitor or angiotensin-receptor blocker (%)

47

Use of a beta-blocker (%)

52

Use of a diuretic (%)

25

Use of mineralocorticoid receptor antagonist (%)

18

Use of digoxin (%)

21

**Supplemental Table 2**. Statistics for Order Effects

| Outcome | p for order effect |
| --- | --- |
|  |  |
| MLWHF score | 0.372 |
| SF-36v2 PCS | * 0.032 |
| LVEDD (cm) | 0.962 |
| LVESD (cm) | 0.596 |
| Peak VO2 (ml/kg/min) | 0.892 |
| NT-Pro BNP (pg/ml) | 0.379 |
